# Gamblers Anonymous as a Recovery Pathway: A Scoping Review

**DOI:** 10.1007/s10899-016-9596-8

**Published:** 2016-04-04

**Authors:** Andrée Schuler, Peter Ferentzy, Nigel E. Turner, Wayne Skinner, Kathryn E. McIsaac, Carolyn P. Ziegler, Flora I. Matheson

**Affiliations:** 1 Centre for Research on Inner City Health, Li Ka Shing Knowledge Institute, St. Michael’s Hospital, Toronto, ON Canada; 2 Centre for Addiction and Mental Health, University of Toronto, Toronto, ON Canada; 3 Dalla Lana School of Public Health, University of Toronto, Toronto, ON Canada; 4 Health Sciences Library, St. Michael’s Hospital, Toronto, ON Canada

**Keywords:** Gambling, Mutual aid, Gamblers Anonymous, 12-step, Problem gambling

## Abstract

Given the preponderance of Gamblers Anonymous (GA), there has been relatively little effort to explore the existing evidence base on its effectiveness as a recovery approach for problem gambling. To remedy this gap in the literature we conducted a scoping review of the literature on mutual aid for individuals experiencing problem gambling published between 2002 and 2015. We searched 13 databases and reviewed reference lists and websites of relevant organizations. We reviewed records for eligibility and extracted relevant data from eligible articles. Three reviewers independently assessed the methodological quality of the included studies using the Mixed Methods Appraisal Tool. We identified 17 studies in 25 publications that were eligible for inclusion. Most studies were conducted in the United States, were cross-sectional in design, and involved both male and female adult participants. Results indicate that the evidence for the effectiveness of GA either as a control condition or in conjunction with formal treatment or medication is inconsistent. An emphasis on patience, using the Serenity Prayer as a way to gain acceptance of financial matters and reality, and absolute assertion of identity as a “compulsive gambler” were identified as important aspects of GA’s recovery culture. There is a need for large-scale randomized controlled trials to determine GA’s effectiveness, as well as research exploring the mechanisms through which GA works, barriers to GA as a recovery approach, and the status of women in the fellowship.

**Electronic supplementary material:**

The online version of this article (doi:10.1007/s10899-016-9596-8) contains supplementary material, which is available to authorized users.

## Introduction

Mutual aid (often called “self-help” and sometimes “peer support”) is a broad designation. At the core, mutual aid is a way to bring people together to address a shared problem, be it cancer, drug use or gambling (Humphreys 2004). In a quasi-experimental study, Humphreys and Moos (2007) examined healthcare costs for patients in a 12-step mutual-aid group and those in cognitive behaviour (CB) programs. Patients in the CB programs used more inpatient and outpatient health services, compared to 12-step group participants, whose health care costs were 30 % less. Involvement in mutual aid has been associated with better results with biological afflictions such as breast cancer (Davison et al. 2000). While the 12 Step model does flourish in mutual aid groups of all kinds, only in recovery from substance use disorders and other addictive behaviours does it dominate so thoroughly (Ferentzy et al. 2004a). In a systematic review of Alcoholics Anonymous (AA), Kelly et al. (2009) found that recovery in AA may stem from the ability of the group processes to augment self-efficacy, coping skills, and motivation, and by helping people build supportive and pro-social networks. According to these authors, AA’s success may lie in its capacity to offer on-going, free, and relatively easy access to common recovery-based therapeutic features, which can be self-regulated based on perceived need.

Founded in the 1950s (Browne 1994; Ferentzy et al. 2004a), Gamblers Anonymous is a mutual aid fellowship based on 12-step principles. GA has meetings in most North American communities, and has established itself worldwide as a resource for people struggling with gambling problems. GA has a unique culture of recovery that, in certain ways, distinguishes it from fellowships such as Alcoholics Anonymous (AA) and Narcotics Anonymous (NA). One feature that makes GA unique is the need to focus on the crippling financial difficulties many individuals experiencing problems with gambling must face. GA devotes much time and energy to counseling members on financial and legal challenges (Browne 1994; Ferentzy et al. 2006b; Ferentzy et al. 2010a).

Despite the lack of involvement of clinicians as treatment providers, GA has long been an accessible option for persons seeking help for a gambling problem, with daily meetings all over Ontario and in most North American cities (Ferentzy et al. 2006a, b; Viets and Miller 1997; Humphreys 2004). An earlier review of the literature on GA (Ferentzy and Skinner 2003) identified several gaps in knowledge, and offered up the following areas for future research: an examination of GA’s effectiveness with larger and varied samples, better accounting of GA beliefs and practices, assessing the performance of GA with formal treatment regimens as well as attendance at other mutual aid organizations, an assessment of who may be best and least suited to GA, and an understanding of how GA can help in the process of recovery. While questions remain about GA’s effectiveness, with many arguing that it might be most helpful in conjunction with formal treatment (Lesieur and Blume 1991; Mark and Lesieur 1992; Petry 2002), the mere fact that meetings are free to attend ensures that GA will continue to have an ongoing role as an adjunct to cash strapped treatment agencies and also as a stand-alone option for many people experiencing problems with gambling. Given the preponderance of GA, there has been relatively little effort to explore the existing evidence base on its effectiveness as a recovery approach for problem gambling. To remedy this gap in the literature we conducted a scoping review of the literature on mutual aid for individuals experiencing problem gambling published between 2002 and 2015. The purpose of the review is to provide a summary of research findings from recent literature on GA, identify gaps in knowledge, and propose specific avenues for future research.

## Methods

We aligned our methodology for the scoping review with the recommendations of Arksey and O’Malley (2005): which includes: identifying the research question; searching for relevant studies; selecting studies; charting the data and collating and summarizing and reporting the results. This approach allowed us to incorporate a range of study designs and address questions beyond those related to treatment efficacy such as descriptions of GA practices and recovery in GA.

### Search Strategy

The following databases were searched: Medline, PsycINFO, Embase, Social Work Abstracts, CINAHL, EBM Reviews, Applied Social Sciences Index and Abstracts (ASSIA), International Bibliography of the Social Sciences (IBSS), ProQuest Dissertations & Theses Global, Social Services Abstracts, Sociological Abstracts, Web of Science, and the Campbell Collaboration Library of Systematic Reviews in June 2015. WorldCat, a global catalog of library collections, was also searched. The search terms included a combination of medical subject headings and keywords for the concepts of gambling and mutual aid, combined with the Boolean operator AND (see Online Resource 1 for search strategy). The search strategy and the systematic search of the literature were developed by an information technician with input from the project team.

We limited the searches to include English language articles and grey literature published between 2002 and June 2015. We reviewed reference lists of included studies. We searched websites of relevant organizations, specifically the Gambling Research Exchange of Ontario (GREO), the Centre for Addiction and Mental Health, and the Alberta Gaming Institute.

A previous review of the literature covered the time period up until 2002 (Ferentzy and Skinner 2003). Consequently for this review, we restricted the time period to papers published from 2002 to 2015. Although the previous work was not a scoping review we feel that it adequately examined earlier papers and that a focus on studies published in the past 13 years (2002–2015) was appropriate.

### Study Selection and Data Extraction

The project team reviewed the titles and abstracts of ten publications from the electronic database search in order to define our eligibility criteria. Studies were included if they involved adults and adolescents who had identified problems with gambling, were attending GA meetings, or were in GA and also in treatment. Eligible study designs included randomized controlled trials, observational studies (cohort, cross-sectional, case–control), descriptive, and qualitative and mixed methods studies. In a cohort study, participants are included based on well-defined characteristics or exposure (or lack of exposure) to a particular factor (Morabia 2004; Song and Chung 2010). The participants are followed over time to determine the occurrence of a disease or some other outcome of interest (Morabia 2004; Song and Chung 2010).We excluded studies where GA was offered embedded in a treatment regimen.

Two team members independently screened the remaining titles and abstracts for eligibility, and any disagreements were resolved by discussion. If there was not enough information to make an informed decision from a title or abstract review, the publication was retrieved and reviewed. For full publication review, two team members conducted a pilot of five publications to ensure a high level of agreement regarding eligibility. One reviewer then reviewed the remaining publications to assess eligibility. Any unclear decisions regarding eligibility were resolved by discussion, and/or consultation with a third author.

### Data Extraction

For eligible publications, one reviewer extracted information regarding study context and design, aims/research questions, participant characteristics, and relevant outcome data/results (e.g., effectiveness of GA, descriptions of GA practices and/or recovery in GA), using a data extraction form that we developed, piloted, and modified.

When the results of a study were reported in more than one publication, we combined the articles and classified the publication with the most complete data as the primary reference; the other publications describing the same study were classified as associated papers (Bunn et al. 2014).

### Quality Assessment

Three reviewers independently assessed the methodological quality of the included studies and resolved disagreements through discussion. We used the Mixed Methods Appraisal Tool (Pluye et al. 2011) which allowed us to assess the quality of diverse study designs including qualitative, mixed-methods, and quantitative studies. The Mixed Methods Appraisal Tool (MMAT) has four criteria to assess the quality of each study design (Pace et al. 2012; Pluye et al. 2011). This tool has been content validated (e.g., items developed from a literature review and consultations and workshops and experts), and has been tested for efficiency and reliability (Pace et al. 2012; Souto et al. 2015). Studies were scored as follows:—(0 % of quality criteria met); * (25 % of quality criteria met); ** (50 % of quality criteria met); *** (75 % of quality criteria met) or **** (100 % of quality criteria met).

## Results

### Description of Included Studies

**Fig. 1:**
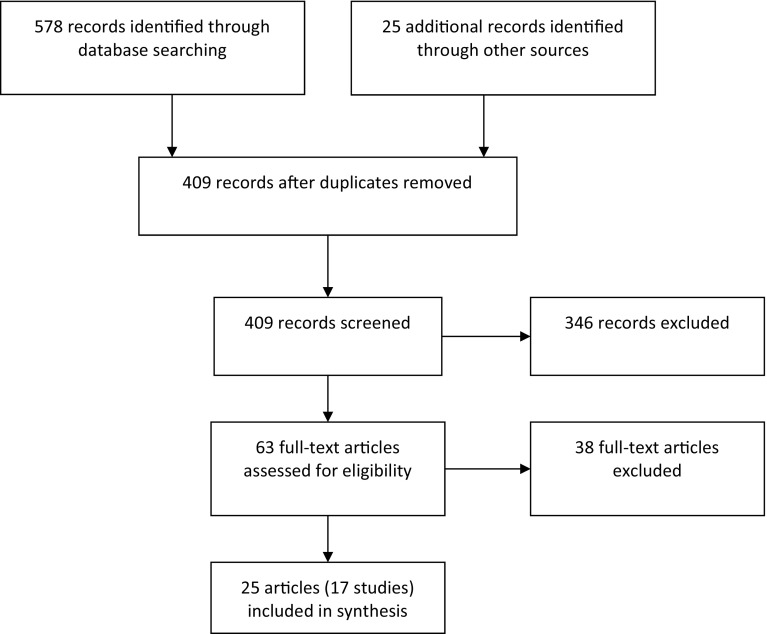
Flow diagram of study selection

As shown in Fig. 1, we identified 603 records: 578 through database searches, 1 from reference lists of eligible publications, and 24 from the grey literature search. After eliminating duplicates, there were 409 records remaining. Of these, 63 were eligible for full review and 25 articles were eligible for inclusion. These 25 articles represent 17 unique studies. Of these 25 publications, 17 studies were classified as primary references (Avery and Davis 2008; Bulcke 2008; Cooper 2003; De Castro et al. 2005; Desai et al. 2012; Ferentzy et al. 2004a, 2007; Gomes and Pascual-Leone 2009; Grant et al. 2009; Grant and Kim 2002; Laracy 2011; Linardatou et al. 2014; Oei and Gordon 2008; Petry 2003; Petry et al. 2006; Toneatto 2008; Straus 2006) and 8 studies (Ferentzy et al. 2004b, 2006a, b, 2009; 2010a, b; Ledgerwood et al. 2007; Petry et al. 2007) as associated publications.

Table 1 summarizes key characteristics of included studies. The majority of studies were conducted in the United States (47 %), 35 % of studies conducted in Canada, and the remaining studies (18 %) were conducted in Australia, Greece, or Brazil. In terms of study design, 29 % of studies were cross-sectional, 24 % were qualitative, 24 % were randomized controlled trials, 18 % were mixed methods, and 6 % were cohort. Fourteen studies included both men and women, two studies included only women, and in one study, the gender composition of the sample was not specified. The majority of studies (88 %) included only adults.

**Table 1:** Characteristics of studies included in scoping review (N = 17)

Characteristic	Number of studies (%)
Country	
USA	8 (47.1)
Canada	6 (35.3)
Australia	1 (5.9)
Greece	1 (5.9)
Brazil	1 (5.9)
Type of study	
Qualitative	4 (23.5)
Cohort	1 (5.9)
Cross sectional	5 (29.4)
Randomized controlled trial	4 (23.5)
Mixed methods	3 (17.7)
Participant gender	
Male	0
Female	2 (11.8)
Both	14 (82.4)
Not specified	1 (5.9)
Participant age group	
Adults	15 (88.2 %)
Youth	0
Both	0
Not specified	2 (11.8)

### Reporting the Results

Studies were grouped into the following categories: (1) randomized controlled trials that evaluated the effectiveness of GA either as a control condition or as an adjunct treatment to medication or psychotherapy, (2) quantitative non randomized studies that (a) described characteristics of individuals who attend GA or (b) examined the association between attending GA and various outcome measures, and (3) qualitative and mixed methods studies that explored GA practices.

### Randomized Controlled Trials that Evaluated the Effectiveness of GA Either as a Control Condition or as an Adjunct Treatment to Medication or Psychotherapy

Four randomized controlled trials have examined the effectiveness of referral to GA either as a control condition or as an adjunct treatment to medication or psychotherapy (Desai et al. 2012; Grant et al. 2009; Linardatou et al. 2014; Petry et al. 2006). Two additional associated papers describing the same study as Petry et al. (2006) were also identified (Ledgerwood et al. 2007; Petry et al. 2007).

In a 12-week trial conducted by Desai et al. (2012), participants (n = 36) were randomized to one of four treatment groups: Bupropion and Harm Reduction, Bupropion and GA, Placebo and Harm Reduction, or Placebo and GA. The authors were not able to detect any significant group differences for any of the outcomes examined (gambling frequency and intensity, treatment compliance, global functioning, disability, and treatment motivation) at study completion. However, when considering the entire sample as a whole, 77 % of the participants showed reductions in the amount of money spent on gambling, 60 % showed reductions in the amount of time spent gambling, and the majority (63–74 %) demonstrated improvements on various measures assessing gambling symptom severity at study completion.

Linardatou et al. (2014) compared the effectiveness of a stress management intervention (n = 23) as an adjunct treatment to GA, compared to GA alone (n = 22) on psychosocial functioning over an 8-week follow-up period. The stress management intervention involved education on diet and exercise, stress coping methods, relaxation breathing and progressive muscle relaxation. The authors found that the stress management program group showed greater reductions in depression, anxiety, and stress, relative to the GA group. Moreover, compared to those in the GA group, the stress management group showed greater improvements in life satisfaction and duration of sleep. No significant differences were observed in the rate of relapse between the two groups.

Grant et al. (2009) randomized participants to either six sessions of imaginal desensitisation plus motivational interviewing (IDMI) (n = 33) or GA (n = 35) over an 8-week period. Individuals initially assigned to the GA group then received six sessions of IDMI, beginning 1 week after the 8-week post-GA assessment. The IDMI group showed significantly greater reductions in gambling symptom severity, depression and anxiety symptoms, as well as psychosocial functioning compared to the GA group over the 8-week treatment period. Moreover, 64 % of participants in the IDMI group were abstinent from all gambling for at least 1 month by the end of the 8-week period, whereas only 17 % of participants in the GA group were abstinent. Men and women did not differ in terms of treatment response. Participants who were initially assigned to GA showed significant improvements in gambling symptoms, psychosocial functioning, and quality of life after receiving IDMI following 8 weeks of GA. Although IDMI was found to be superior to GA in improving PG symptoms and other outcomes, the authors acknowledge that individuals assigned to GA attended meetings very infrequently (mean weekly attendance 1.1 meetings), which precluded the possibility of ruling out whether GA would be more effective if attended more regularly.

Petry et al. (2006) randomized participants to referral to GA (n = 63), GA referral plus a cognitive–behavioral (CB) workbook (n = 84), or GA referral plus 8 sessions of individual cognitive behavioural therapy (CBT) (n = 84) over a 2-month treatment period. Participants in the CB workbook and CBT conditions showed greater improvements in gambling symptoms, and reductions in days gambled to a greater extent over the 2-month treatment period than those in GA referral alone. The number of GA meetings attended by the 2-month posttreatment visit did not differ across treatment groups. Participants in the GA referral, workbook, and therapy conditions, respectively attended 1.7, 2.1, and 2.3 GA meetings during the 2-month treatment period. At the 12-month follow-up visit, mean number of meetings attended were 7.6, 6.8, and 7.4. However, at the last follow-up conducted for each participant, about half the participants (41.3, 38.1, and 53.6 % in the three groups) never attended any GA meetings during the year. The number of GA meetings attended was positively associated with past-month gambling abstinence at the posttreatment evaluation and the 12-month follow-up. At the 12-month follow-up visit, participants in the CB workbook and CBT arms continued to show greater improvements in gambling symptom scores, but not in days gambled than those in GA referral alone. At 12-months, proportions of participants abstinent, substantially reduced, somewhat reduced, or no change did not differ by group; percentages classified as abstinent or substantially reduced gambling were 60.5, 60.0, and 65.7 % in the GA referral, CB workbook, and CB therapy conditions, respectively.

In an associated study to Petry et al. (2006), only focusing on participants in the CBT + GA referral group and the GA referral alone group, Petry et al. (2007) examined whether coping skills acquisition mediated the effects of CBT on reducing gambling. In the overall sample, coping responses improved between baseline and the posttreatment evaluation, with participants in the CBT group demonstrating greater coping skills than those assigned to the GA referral alone condition. Changes in coping skills scores mediated the relationship between treatment assignment and gambling outcomes from pretreatment to posttreatment, but mediation effects were not as robust throughout the 12-month follow-up period.

In another associated study to Petry et al. (2006), Ledgerwood et al. (2007) assessed differences in baseline characteristics and treatment outcomes among individuals who reported committing gambling-related illegal acts (n = 63) and those who reported that they did not commit any gambling-related illegal acts (n = 168) in the year prior to treatment. Compared to those without a recent history of gambling-related illegal behaviour, participants with a recent history of gambling-related illegal behavior were significantly younger, had more severe gambling symptoms at intake, had more gambling debt, and were more likely to meet criteria for anti-social personality disorder. Reductions in gambling symptoms were observed throughout both the treatment and follow-up periods, and were higher among those with recent histories of gambling-related illegal behaviour compared to those without. Participants with and without gambling-related illegal behavior did not differ in terms of improvement in gambling outcomes over time.

### Quantitative Non-randomized Studies that Described Characteristics of Individuals Who Attend GA or Examined the Association Between Attending GA and Various Outcome Measures

Two studies described characteristics of individuals who attended GA (Petry 2003; Toneatto 2008). Petry (2003) compared people identified with pathological gambling who had experience with GA to those who had not previously attended GA. Both groups were entering professional treatment for pathological gambling. While equal proportions were female (40 %) and members of ethnic minorities (13 %), GA attendees had higher incomes than non-GA attendees, whereas participants who were single were less likely to attend GA than those who were married or divorced. Compared to those who had never attended GA, those who attended GA had higher gambling symptom scores, a longer duration of gambling problems, and a larger gambling debt. GA attendees gambled fewer days in the month prior to entering treatment, and were more interested in receiving gambling treatment. No group differences were observed for age of gambling initiation or amount gambled in the month prior to beginning professional treatment. Participants who attended GA before initiating professional treatment had fewer current drug problems (although both groups had low Addiction Severity Index drug composite scores), but more severe family/social problems. GA attendees and non-attendees were equally likely to have received professional treatment for a substance use disorder, or to have ever attended a self-help meeting for substance abuse. However, compared to non-GA attendees, individuals who had attended a GA meeting were less likely to be involved in treatment for a substance use disorder currently, and to have used illegal drugs in the past month. GA attendees reported being more bothered by family problems. Moreover, participants with a history of GA attendance were more likely to become reengaged with GA during the 2 months after beginning professional treatment, and were more likely to become actively involved in professional treatment. Finally, individuals with a history of GA attendance were more likely to be abstinent from gambling 2 months after beginning professional treatment.

Toneatto (2008) assessed the reliability and validity of the Twenty Questions (20Q) a screening instrument for problem gambling in three independent samples: two samples in treatment (n = 61, n = 99, respectively) and one sample not receiving treatment (n = 296). The 20Q was highly correlated with the DSM-IV (American Psychiatric Association 2000) indicating that it is a valid measure of problem gambling. Toneatto (2008) also conducted cross-sectional analyses to describe the association between the 20Q and gambling treatment history, including attendance at GA and other treatment sessions. GA attenders had significantly higher gambling severity scores (20Q scores) than those who had never attended GA in all three independent samples, but the GA 20Q was not correlated significantly with the number of GA sessions attended or with the number of treatment sessions attended (Toneatto 2008).

Four studies examined the association between attending GA and various outcome measures (De Castro et al. 2005; Gomes and Pascual-Leone 2009; Grant and Kim 2002; Oei and Gordon 2008). De Castro et al. (2005) examined the association between treatment condition (weekly GA meetings, clinical treatment, or both) and all items of the Gambling Follow-Up Scale (GFS), a measure for follow-up and treatment outcome for individuals experiencing problem gambling issues. The three treatment conditions did not differ in terms of frequency and time spent gambling, work status, or family relationship. However, participants enrolled in GA (GA only or GA + clinical treatment) had better scores on the leisure item of the GFS (“How have you occupied your free time in the last 4 weeks? Do you take initiatives to keep yourself busy? Are you satisfied with using your free time this way?”) compared to participants receiving clinical treatment only.

In a secondary analysis of a cohort of participants enrolled in two different randomized controlled trials (n = 131), Grant and Kim (2002) examined gender differences in terms of demographic characteristics, problem gambling symptoms, and treatment history among participants experiencing pathological gambling issues seeking treatment. The majority of men (59.0 %) and women (68.1 %) with a treatment history of GA did not report a response to treatment (full remission of symptoms, moderate improvement, slight improvement, no change, worsening of symptoms) to either GA or outpatient treatment, nor were there any significant differences observed between men and women in terms of self-reported response to treatment to GA with respect to gambling behavior and urges.

Gomes and Pascual-Leone (2009) explored the change facilitating effects of certain characteristics or conditions of an individual being treated for problem gambling: emotional support, instrumental support, emotional awareness, GA involvement, and depressed affect among 60 outpatients. The authors found that GA involvement was associated with readiness for change, but was not associated with abstinence self-efficacy, motivation for change, or gambling severity.

Oei and Gordon (2008) examined the factors that differentiated abstinent and relapsed individuals among GA members (n = 75). They found that GA member’s attendance and participation in meetings, adherence to the 12-steps to recovery program adopted by GA, belief in God, belief in a higher power, and support from family and friends had a positive impact on their gambling abstinence. Although belief in God and belief in a higher power significantly predicted group membership (abstinent vs. relapsed), this was not as important as GA attendance and participation in meetings. Increased gambling urges and erroneous cognitions increased the chance of relapse.

### Qualitative and Mixed Methods Studies that Explored GA Practices

Avery and Davis (2008) explored recovery approaches and patterns specific to women (n = 136). 12-step participation was a common theme among those in recovery. Seventy-five percent of the women attended at least one GA meeting. Nearly three quarters of the women who answered the questions pertaining to GA (n = 86; 72 %) reported that they still attended GA meetings. Many current members (n = 55) reported sponsoring others in GA. For the women who attended GA meetings, frequent attendance was typical in early recovery. They reported that when attending GA meetings, they felt welcomed and understood by individuals experiencing the same problems, no longer felt alone, felt they could tell the truth, and that “meetings instilled hope that problems could be solved” (p. 181). However, some participants indicated that they felt unwelcome at GA meetings because of being a woman. Moreover, issues with how GA emphasized abstinence and finding the group to be “unsympathetic” were also reported. Only 8 % (n = 7) had relatives or friends attending GamAnon. GamAnon was viewed as helpful during the recovery process by some participants as it helped loved ones understand problem gambling and their own role in assisting the recovery process. However, GamAnon was seen as unhelpful during the recovery process by other participants because of the lack of program facilitation and because some family members or friends used GamAnon to increase their sense of “righteousness”.

Bulcke (2008) compared the treatment barriers identified by women involved and not involved with GA. The treatment barriers ‘gamble to deal with the stress of my daily life,’ (socio-environmental factor) ‘feeling ashamed to admit I have a gambling problem,’ (individual issue/personal beliefs, feelings and thoughts) and ‘feeling lonely without gambling in my life,’ (individual issue) were reported most often by both groups. In contrast, the individual barrier item (personal beliefs, feelings and thoughts) ‘raised to believe I can take care of my own problems’ was identified by over 20 % of those respondents involved in GA. It was not, however, identified at all (0 %) by respondents who were not involved in GA. Ninety-three percent of participants reported prior involvement with GA. Women involved with GA did not differ from women not involved with GA on any of the treatment barrier subscale scores (treatment program characteristics; personal beliefs, feelings and thoughts; and socio-environmental factors).

Straus (2006) investigated the process and effects of direct member-to-member comments at GA meetings through the use of surveys and telephone interviews. Often derided (and prohibited) as “cross-talk” in the 12 Step world, many (though not all) GA meetings allow and even encourage members to comment on what other members have shared. Despite a few misgivings by some members, overall comments were considered beneficial by members with respect to support for not gambling and insight into “characterological issues” (p. 75). Straus (2006) identified that GA members found the following benefits of GA attendance: feelings of belonging, a commitment to abstinence, “instillation of hope, social learning, and altruism” (p. 103). One potential drawback offered involved disclosure (or the lack of it): “It seems plausible that the prospect of receiving comments at a GA meeting could influence what members choose to share during their therapy. There is clearly the possibility that others’ judgments of them will be conveyed through comments” (p. 100).

Laracy (2011) explored the experiences of both current and past GA members. The author found that for current members, most participants felt they were unable to discuss their gambling issues with family and friends, and that GA provided a safe forum “for self-disclosure and discussion” (p. 121) as other members shared similar experiences. By following the lead of veteran members, new members learned to utilize the medical terminology used in the program to understand and realize that they were “compulsive gamblers” (e.g., self-identify as someone experiencing problems with gambling). Moreover, by attending GA meetings and achieving a greater understanding of their gambling behaviour, “the compulsive gambler identity becomes the dominant or master status in one’s self-image” (p. 123) and can be considered a key component in recovery. That is, in GA it is believed that one has to accept the identity of “compulsive gambler” for the rest of one’s life. Past members’ indicated they that no longer attended GA meetings due to the religious undertones of the program, while others indicated that identifying as a “compulsive gambler” for the rest of their lives was not a positive approach to recovery. The author found that GA also provided a way for women to establish an informal social network with other women who attend GA meetings (connecting via phone calls, coffee dates, etc.) which seemed more important for women’s recovery, and proposed that this could be why few women stay in GA. Finally, many former GA members preferred addiction services counseling for gambling rather than GA, because of “leadership by a trained professional and an ability to discuss other underlying problems” (p. 126) that “may or may not have resulted in an excessive gambling habit” (p. 127) (e.g., other mental health problems, relationship issues/problems). Moreover, former GA members preferred these counselor-led sessions to GA because they placed more of an emphasis on hope and eventually returning to a normal life, and counselor-led programs do not expect one to identify oneself as a “compulsive gambler” for the rest of one’s life (Laracy 2011).

Cooper (2003) explored reasons individuals avoided self-help groups such as GA and/or specialist treatment services for gambling issues. The majority of the sample reported that they avoided any form of treatment (GA or other specialized services) because of stigma, including concerns regarding others’ opinions and disclosing personal information. The mean number of stigma scores (total number of reasons for avoiding GA and/or treatment) was 2.16 for those who attended GA but not treatment, 2.50 for those who attend GA + treatment, and 4.22 for those who had not received any care, with a significantly lower stigma score for those who attended GA-only relative to those who did not receive any care. Those who reported that their GA affiliations were “extensive” had significantly lower stigma scores than non-extensive GA attendees and those who did not receive any form of treatment.

Ferentzy et al. wrote a series of articles about GA practices (the ways in which GA members pursue recovery) with an emphasis on how GA has a recovery culture that is unique among 12 Step fellowships. Members’ beliefs and attitudes about recovery were also described. These authors conducted two qualitative/ethnographic studies of GA. The first (Ferentzy et al. 2004a) involved participant observation at 42 GA meetings and 23 semi-structured interviews with GA members in southern Ontario. In this study, the age of interviewees ranged from 26 to 70+ years, with a male–female ratio of 15 males to 8 females. Time abstinent from gambling ranged from 1 week to 35 years. The second (Ferentzy et al. 2007) involved semi-structured interviews with 39 GA members from various North American locales. Participants were 35 to 70+ years, with a male–female ratio of 26 males to 13 females. Information on time abstinent from gambling was not available.

In the first study, Ferentzy and colleagues (Ferentzy et al. 2004a, b, 2006a, b), found that GA had undergone noteworthy changes over the previous 10–20 years in both Canada and the US. Previous studies had indicated that GA had for the most part ignored the 12 Steps (Browne 1991, 1994) as well as emotional issues, in favor of a recovery culture that focused almost exclusively on abstinence from gambling and practical matters such as debt (Browne 1991, 1994). With their study, Ferentzy et al. found that GA had become more focused on the 12 Steps and that members were now encouraged to discuss emotions and other life issues (Ferentzy et al. 2004a, 2006a).

Ferentzy et al. also observed that GA had long had a reputation as male centered (Ferentzy et al. 2004a, b), with very few women and a recovery culture that downplayed women’s needs and concerns (see for example: Mark and Lesieur (1992)). A notable example would be the preponderance of “war stories”—recovery jargon for graphic and often disturbing accounts of one’s life in active addiction—said to alienate many women. However, Ferentzy et al. found that the number of women in the Toronto area was then about 20 % and rising, war stories were less common, and other changes amenable to women had also taken place (Ferentzy et al. 2004a, b). For example, while many of the older GA members often downplayed the significance of gambling options pursued more frequently by women (e.g., bingo), this was also changing. Ferentzy et al. (2004a, b) referred to earlier work done by Crisp et al. (2000), who found that men were more likely to report “external concerns” (employment, legal) as important whereas women were more likely to report concerns with “physical and interpersonal issues” such as health concerns and improving family ties. Ferentzy et al. noted that GA had become much more amenable to monologues related to interpersonal and other issues important to women (Ferentzy et al. 2004a, b).

Though GA is modeled after AA, Ferentzy and his colleagues described GA’s very different approach to the 12 Steps of recovery, and also examined many circumstances specific to gambling by way of explanation (Ferentzy et al. 2006b). One notable feature of GA identified was an emphasis on patience in the recovery process—even the 12 Steps themselves may be practiced very slowly (e.g., members may take time to work through all of the steps) in GA (Ferentzy et al. 2006b). Ferentzy et al. (2006b) identified one key aspect of gambling responsible for this emphasis on patience. While a person with a substance use disorder must be on guard against the instant gratification offered by alcohol or drugs (i.e., the high) and must learn to forgo instant gratification (i.e., patience), a person with gambling problems must also forgo similar need for instant gratification and, beyond that, must sacrifice the potential for solving many problems quickly by winning some money. So an even keener emphasis on patience—not trying to solve problems quickly—is an important aspect of recovery in the GA culture. This finding emerged from the in-depth interviews as well as participant observations, and was also apparent in GA’s main text (Gamblers Anonymous International Service Office (GAISO) 1999).

The second qualitative study (Ferentzy et al. 2007), involving interviews with 39 GA members from various regions of Canada and the US, also found signs of the unique recovery culture in GA that sets it apart from other 12 step fellowships (Ferentzy et al. 2007, 2009, 2010a, b). The authors note, for example, that GA’s fourth step involves a financial as well as a moral inventory—an innovation unique to GA [Gamblers Anonymous International Service Office (GAISO) 1999]. As well, even if members wish to “work” the 12 Steps, in practice the original sequence in order from step one to step twelve is not suitable for many GA members. Steps which have a direct bearing on financial matters (e.g., four and nine) often come first (Ferentzy et al. 2009) because of the huge financial problems faced by many individuals experiencing problems with gambling. Thus the 12-steps may not be followed in the same order as they are with AA and NA.

Another major way in which GA differs from other 12 Step fellowships is that GA fosters a “socially based conception of recovery” that relies on family members, including significant others, for social support. This state of affairs stems from the involvement of GA’s sister fellowship, GamAnon (Ferentzy et al. 2010a), intended for anyone who is affected by a loved one’s gambling. In practice GamAnon is mainly composed of wives of male GA members (Ferentzy et al. 2010a). Typically, GamAnon meetings are held in settings geographically close to GA meetings (e.g., next door). Many GA members insist that their wives are their “sponsors,” something anathema to traditional 12 Step recovery where a sponsor should be a peer (with commensurate alcohol or drug use disorders) (Ferentzy et al. 2010a). Again, these authors observed that financial issues help to explain this situation. For one, family fortunes are not private matters so pecuniary issues engendered an approach that, overall, is unique to GA. Family members, typically wives of male GA members, have an obvious stake in financial matters pertaining to their spouses gambling and are on guard against behaviors that could, for example, cause them to lose their homes. Ferentzy et al. (2010b) even note that GA, being privy to the importance of spousal support was, already in the mid-20th century, well ahead of the treatment and research communities. But these authors clarify that, overall, GA is not more social or family centered than other 12 Step fellowships. With many members often guarded, private and who arguably want a sense of independence to manage their recovery, this one aspect of GA involving the input of spouses is an anomaly in an otherwise hyper-masculine recovery culture (Ferentzy et al. 2006b, 2010a).

Ferentzy et al. (2010b) also identify the role of the Serenity Prayer as reflective of GA’s recovery culture: “God grant me the serenity to accept the things I cannot change, courage to change the things I can, and the wisdom to know the difference” [Gamblers Anonymous International Service Office (GAISO) 1999]. Despite being focused more on money matters, with the help of a prayer such as this one, members (even atheists) may repeat it over 100 times daily, and achieve more than financial freedom: honesty, acceptance, and humility can be learned this way, even if the pivot is money (Ferentzy et al. 2010b). While GA members use this prayer to gain acceptance of, and clarity on, money matters, the same prayer can help with a greater overall acceptance of reality, spousal relations, parenting, work relations and friendships (Ferentzy et al. 2010b).

### Methodological Quality of Included Studies

There was considerable variation in the methodological quality of included studies (see Online Resource 2), with scores ranging from—(0 of 4 criteria met) to **** (4 of 4 criteria met). Randomized controlled trials were of low to moderate quality, quantitative non-randomized studies were all of moderate to high quality. The majorities of qualitative studies (75 %) were of high quality, and mixed methods studies were all of low quality.

## Discussion

This review of the literature on Gamblers Anonymous identified 25 publications representing 17 unique studies that were published between 2002 and 2015. The majority of studies were conducted in the United States, were cross-sectional in design, and involved both male and female adult participants. The greatest number of studies reviewed (n = 7) focused on providing insight into GA practices (the ways in which GA members pursue recovery) and potential features of GA that may impact recovery. Fewer studies evaluated the effectiveness of GA as a recovery approach for problem gambling (n = 4), the association between GA attendance and various outcome measures (n = 4), or described characteristics of individuals attending GA (n = 2).

A previous review of GA (Ferentzy and Skinner 2003) indicated that large-scale, controlled studies that examined the efficacy of GA (alone and as an adjunct treatment to other interventions) were needed. Very few randomized controlled trials assessing the effectiveness of GA as an intervention for gambling were identified in the current scoping review. These studies reported mixed findings regarding the effectiveness of GA either as a control condition or in conjunction with formal treatment or medication. One study found that GA as adjunctive treatment to pharmacological intervention did not result in significantly different gambling outcomes than other forms of treatment (Desai et al. 2012); another showed that cognitive behavioural interventions alone and in conjunction with GA were more effective in improving gambling outcomes relative to GA referral alone (Petry et al. 2006); and finally, one study reported that imaginal desensitization plus motivational interviewing was superior to GA referral in reducing gambling behaviours (Grant et al. 2009). Preliminary research also indicates that stress management intervention may be an effective adjunctive treatment to GA in improving other non-gambling related outcomes including stress, anxiety, depression, sleep quality and life satisfaction, but not relapse rates (Linardatou et al. 2014). Future research assessing the efficacy of GA would benefit from recruiting larger samples and from evaluating the impact of attendance (number of GA meetings attended) on GA outcomes to determine whether GA would be more effective if attended more regularly.

Given the high levels of comorbid substance use disorders and mental illness among individuals experiencing problem gambling issues (Lorains et al. 2011), it is also possible that single focus mutual aid fellowships such as GA may often be ineffective because of the complex needs of many members of this population. Indeed, a growing body of evidence has documented the effectiveness of dual focus fellowships in improving outcomes among individuals with co-occurring mental health and substance use disorders (Magura et al. 2002, 2003; Rosenblum et al. 2014). There are in fact multi-focus 12-step organizations such as Addictions Victorious and All Addictions Anonymous that focus on multiple types of addictions. However, the effectiveness of these mutual aid organizations in improving outcomes among individuals experiencing problem gambling and concurrent addictions or mental illness is unknown.

This review suggests that individuals who are involved with GA may differ from individuals who are not involved with GA on various domains. GA attendees have more severe gambling symptoms and are more motivated to receive gambling treatment (Petry 2003). Moreover, a history of GA attendance appears to be associated with greater subsequent involvement with professional treatment (Petry 2003). Consistent with prior research predating the time period covered in this review suggesting that GA is positively associated with improvements in abstinence (Lesieur and Blume 1991; Stewart and Brown 1988), our review also indicates that GA involvement is associated with abstinence (Oei and Gordon 2008; Petry 2003), increased readiness for change (Gomes and Pascual-Leone 2009), increases in coping skills (Petry et al. 2007), and higher levels of leisure activity (De Castro et al. 2005). It is not possible to draw any firm conclusions regarding the causal relationship between GA involvement and treatment outcomes because the majority of studies that showed associations between GA and positive outcomes were either cross-sectional or cohort studies.

A previous review highlighted the need for more detailed accounts of GA beliefs and practices as well as an enhanced understanding of what GA was able to offer those individuals that it seems to have helped (Ferentzy and Skinner 2003). The authors found an emerging body of research addressing this topic. An emphasis on patience (Ferentzy et al. 2006a), using the Serenity Prayer as a way to gain acceptance of financial matters and reality (Ferentzy et al. 2010b), and absolute assertion of identity as a “compulsive gambler” (Laracy 2011) were identified as important aspects of GA’s recovery culture. GA was seen as helpful in the recovery process because it provided a safe environment for self-disclosure, members felt understood by others undergoing a shared experience, and the meetings instilled hope that gambling issues could be resolved (Avery and Davis 2008; Laracy 2011; Straus 2006). There is a need for more research exploring the mechanisms through which GA works. For instance, little is known about the role of social identity in recovery, and how this mutual identification can increase self-efficacy (an individual’s belief of their ability or inability to succeed in attaining specific goals) (Buckingham et al. 2013). Furthermore, GA’s collective experience can mitigate differences between people based on race, economic class and other potential social barriers. Ferentzy and Skinner (2008) discuss some of the evidence for how the supposed need to abstain completely from gambling involves not only a proscription but also a statement of shared identity: when someone abstains it involves more than just recovery: that person becomes a member of a homogenous collective, part of the GA subculture, with growing seniority and respect as time abstinent increases.

This review also provides preliminary evidence of the status of women in the fellowship. Women in GA appear to participate actively in the fellowship by sponsoring other members, and attending meetings frequently during the early stages of recovery (Avery and Davis 2008). However, some women indicated that they felt unwelcome at GA meetings because of their gender (Avery and Davis 2008). GA has long been identified as a predominantly male domain wherein women’s concerns are often sidelined (Ferentzy and Skinner 2003; Mark and Lesieur 1992). Evidence suggests strongly that this has been changing, due in part to the rise in female problem gambling rates in the wake of legalized gaming venues and, as well, to shifting attitudes about gender roles throughout North America (Ferentzy et al. 2004b, 2010a). Further studies that explore the changing status of women in the fellowship are needed, especially qualitative studies that fully explore women’s lived experience of GA. We also found preliminary evidence that women have used GA to develop informal networks, and that these informal social networks may be more important for women’s recovery (Laracy 2011). Attending GA meetings provides women with networking opportunities where they can gain support in small groups. This move to informal networking may relate to the male-centric nature of GA, and may be one of the reasons that few women stay in GA (Laracy 2011). Overall, there is a lack of research on informal social networks established through GA among persons who no longer attend. GA, even among persons who no longer attend, may reverberate by means of social networks/friendships established during time spent in GA, and more research is needed to determine the significance of these networks and whether they are an essential aspect of ongoing recovery.

This review highlights that few studies have identified barriers to GA as a recovery approach among men and women (Bulcke 2008; Cooper 2003). This topic requires further investigation as any perceived barriers limit an individual’s ability to access treatments for gambling, including GA. There is also a current lack of research regarding the status of GamAnon (e.g., is it vanishing, going strong, or changing?) as well as GamAnon’s role in the recovery of individuals experiencing problem gambling issues (Ferentzy et al. 2010a). The effectiveness of GA compared to natural recovery (recovery without help) also remains to be determined. Still missing in the field is knowledge of the ways in which GA attendance interacts with formal treatment and a better understanding of the profiles of individuals best (and least) suited to GA. These were gaps identified in the earlier review by Ferentzy and Skinner (2003), which still exist.

A limitation of the current review was the inclusion of studies that had GA in a trial arm. Given that the literature searches yielded very few studies which examined the impact of GA on various outcome measures among participants experiencing problem gambling we elected to include any study that had GA in a trial arm, even if referral to GA was a control condition or an adjunct treatment to medication or psychotherapy. While these studies cannot tell us about the actual efficacy of GA, the statistical tests examine the hypothesis that the experimental condition is different from the control (in this case GA), allowing us to explore when and if GA was statistically similar or different from other treatment approaches. At least then we know that treatment was no better than GA.

## Conclusions

GA is a cost-effective and widely available resource for individuals experiencing problem gambling issues, and is an accessible treatment option [perhaps more notably for people of low income and those with gambling-related debt (Ferentzy et al. 2007)]. This scoping review contributes to the existing literature on mutual aid for problem gambling by synthesizing recent literature on GA and providing a comprehensive overview of the evidence base for this mutual aid fellowship as a recovery approach. Finally, this review identified gaps in knowledge and specific avenues for future research.

## Electronic supplementary material

Below is the link to the electronic supplementary material.

Supplementary material 1 (DOCX 22 kb)

Supplementary material 2 (DOCX 29 kb)
