# Supplementary material for: Gamblers Anonymous as a Recovery Pathway: A Scoping Review
Source: J Gambl Stud. 2016 Apr 4;32(4):1261–78. doi: 10.1007/s10899-016-9596-8 (PMC5101261; doi:10.1007/s10899-016-9596-8)
Supplement: Supplementary file 1 — Supplementary material 1 (DOCX 22 kb) [file 10899_2016_9596_MOESM1_ESM.docx]

**Additional file 1. Search strategy**

| **Database(s)** | **Search Terms** | |
| --- | --- | --- |
| Ovid MEDLINE(R) | 1 | Gamblers Anonymous.tw. |
|  | 2 | Gambling/ |
|  | 3 | (gamble* or gambling).tw. |
|  | 4 | (sports betting or slot machine* or video lottery terminal*).tw. |
|  | 5 | 2 or 3 or 4 |
|  | 6 | peer group/ |
|  | 7 | Self-Help Groups/ |
|  | 8 | (mutual aid or mutual support or peer support or 12-Step group* or 12-Step program* or Twelve-step program* or Twelve-step group* or self help group* or support group* or 12 steps or twelve steps or group support* or recovery group* or nonprofessional support or non-professional support or peer to peer or peer counsel*).tw. |
|  | 9 | natural recovery.tw. |
|  | 10 | self-recovery.tw. |
|  | 11 | 6 or 7 or 8 or 9 or 10 |
|  | 12 | 5 and 11 |
|  | 13 | ((gambling or gambler*) adj3 (recovery or recovering)).tw. |
|  | 14 | 1 or 12 or 13 |
|  | 15 | limit 14 to yr="2002 -Current" |
|  | 16 | limit 15 to english language |
|  | 17 | remove duplicates from 16 |
| PsycINFO | 1 | Gamblers Anonymous.ti,ab. |
|  | 2 | ((gambling or gambler*) adj3 (recovery or recovering)).ti,ab. |
|  | 3 | exp gambling/ |
|  | 4 | (gamble* or gambling).ti,ab. |
|  | 5 | (sports betting or slot machine* or video lottery terminal*).ti,ab. |
|  | 6 | 3 or 4 or 5 |
|  | 7 | (mutual aid or mutual support or peer support or 12-Step group* or 12-Step program* or Twelve-step program* or Twelve-step group* or self help group* or support group* or 12 steps or twelve steps or group support* or recovery group* or nonprofessional support or non-professional support or peer to peer or peer counsel*).ti,ab. |
|  | 8 | natural recovery.ti,ab. |
|  | 9 | self-recovery.ti,ab.) |
|  | 10 | exp Peer Counseling/ |
|  | 11 | twelve step programs/ |
|  | 12 | support groups/ |
|  | 13 | self help techniques/ |
|  | 14 | exp "Recovery (Disorders)"/ |
|  | 15 | 7 or 8 or 9 or 10 or 11 or 12 or 13 or 14 |
|  | 16 | 6 and 15 |
|  | 17 | 1 or 2 or 16 |
|  | 18 | limit 17 to english language |
|  | 19 | remove duplicates from 18 |
| Embase | 1 | Gamblers Anonymous.ti,ab. |
|  | 2 | ((gambling or gambler*) adj3 (recovery or recovering)).ti,ab. |
|  | 3 | exp gambling/ |
|  | 4 | (gamble* or gambling).ti,ab. |
|  | 5 | (sports betting or slot machine* or video lottery terminal*).ti,ab. |
|  | 6 | 3 or 4 or 5 |
|  | 7 | (mutual aid or mutual support or peer support or 12-Step group* or 12-Step program* or Twelve-step program* or Twelve-step group* or self help group* or support group* or 12 steps or twelve steps or group support* or recovery group* or nonprofessional support or non-professional support or peer to peer or peer counsel*).ti,ab. |
|  | 8 | natural recovery.ti,ab. |
|  | 9 | self-recovery.ti,ab. |
|  | 10 | exp self help/ |
|  | 11 | exp peer counseling/ |
|  | 12 | exp support group/ |
|  | 13 | 7 or 8 or 9 or 10 or 11 or 12 |
|  | 14 | 6 and 13 |
|  | 15 | 1 or 2 or 14 |
|  | 16 | limit 15 to (english language and yr="2002 -Current") |
|  | 17 | limit 16 to embase |
|  | 18 | remove duplicates from 17 |
| Social Work Abstracts | 1 | Gamblers Anonymous.ti,ab. |
|  | 2 | ((gambling or gambler*) adj3 (recovery or recovering)).ti,ab. |
|  | 3 | (gamble* or gambling).ti,ab. |
|  | 4 | (sports betting or slot machine* or video lottery terminal*).ti,ab. |
|  | 5 | (mutual aid or mutual support or peer support or 12-Step group* or 12-Step program* or Twelve-step program* or Twelve-step group* or self help group* or support group* or 12 steps or twelve steps or group support* or recovery group* or nonprofessional support or non-professional support or peer to peer or peer counsel*).ti,ab. |
|  | 6 | natural recovery.ti,ab. |
|  | 7 | (3 or 4) and (5 or 6) |
|  | 8 | 1 or 2 or 7 (12) |
|  | 9 | limit 8 to yr="2002 -Current" |
| CINAHL |  | (MH "Peer Counseling") OR (MH "Peer Assistance Programs")  (MH "Support Groups")  (mutual aid or mutual support or peer support or 12-Step group* or 12-Step program* or Twelve-step program* or Twelve-step group* or self help group* or support group* or 12 steps or twelve steps or group support* or recovery group* or nonprofessional support or non-professional support or peer to peer or peer counsel* or natural recovery)  gamble* or gambling  (MH "Gambling")  (gambling or gambler*) N3 (recovery or recovering)  "gamblers anonymous"  Limiters - Published Date: 20020101-20151231; English Language |
| EBM Reviews - Cochrane Database of Systematic Reviews, EBM Reviews - ACP Journal Club, EBM Reviews - Database of Abstracts of Reviews of Effects, EBM Reviews - Cochrane Central Register of Controlled Trials, EBM Reviews - Cochrane Methodology Register, EBM Reviews - Health Technology Assessment, EBM Reviews - NHS Economic Evaluation Database | 1 | Gamblers Anonymous.ti,ab. |
|  | 2 | ((gambling or gambler*) adj3 (recovery or recovering)).ti,ab. |
|  | 3 | gambling/ |
|  | 4 | (gamble* or gambling).ti,ab. |
|  | 5 | 3 or 4 |
|  | 6 | (mutual aid or mutual support or peer support or 12-Step group* or 12-Step program* or Twelve-step program* or Twelve-step group* or self help group* or support group* or 12 steps or twelve steps or group support* or recovery group* or nonprofessional support or non-professional support or peer to peer or peer counsel* or natural recovery or self recovery).ti,ab. |
|  | 7 | Self-Help Groups/ |
|  | 8 | 6 or 7 |
|  | 9 | 5 and 8 (8) |
|  | 10 | 1 or 2 or 9 |
|  | 11 | remove duplicates from 10 |
|  | 12 | limit 11 to english language [Limit not valid in CDSR,ACP Journal Club,DARE,CLCMR; records were retained] |
|  | 13 | limit 12 to yr="2002 -Current" [Limit not valid in DARE; records were retained] |
| ProQuest: Applied Social Sciences Index and Abstracts (ASSIA), International Bibliography of the Social Sciences (IBSS), ProQuest Dissertations & Theses Global, Social Services Abstracts, Sociological Abstracts | 1 | all("Gamblers Anonymous" OR "gambling recovery") AND la.exact("English") AND pd(>20020101) |
|  | 2 | all((gamble* OR gambling)) AND all(("mutual aid" OR "mutual support" OR "peer support" OR "12-Step" OR "Twelve-step" OR "self help group" OR "support group" OR "group support" OR "recovery group" OR "nonprofessional support" OR "non-professional support" OR "peer to peer" OR "peer counselling" OR "natural recovery" OR "self recovery" OR "self help groups" OR "support groups" OR "recovery groups" OR "peer counseling" OR "peer counsellor" OR "peer counselor" OR "peer counsellors" OR "peer counselors")) AND la.exact("English") AND pd(>20020101) |
|  | 3 | 1 or 2 |
| Web of Science: Social Sciences Citation Index (SSCI) --1956-present  Conference Proceedings Citation Index- Science (CPCI-S) --1990-present  Conference Proceedings Citation Index- Social Science & Humanities (CPCI-SSH) --1990-present | 1 | ("Gamblers Anonymous" or "gambling recovery")  Indexes=SSCI, CPCI-S, CPCI-SSH Timespan=2002-2015 |
|  | 2 | ((gamble* or gambling)) AND TOPIC: (("mutual aid" or "mutual support" or "peer support" or "12-Step" or "Twelve-step" or "self help group" or "support group" or "group support" or "recovery group" or "nonprofessional support" or "non-professional support" or "peer to peer" or "peer counselling" or "natural recovery" or "self recovery" or "self help groups" or "support groups" or "recovery groups" or "peer counseling" or "peer counsellor" or "peer counselor" or "peer counsellors" or "peer counselors"))  Indexes=SSCI, CPCI-S, CPCI-SSH Timespan=2002-2015 |
|  | 3 | #2 OR #1  Indexes=SSCI, CPCI-S, CPCI-SSH Timespan=2002-2015 |
|  | 4 | #2 OR #1  Refined by: LANGUAGES: ( ENGLISH )  Indexes=SSCI, CPCI-S, CPCI-SSH Timespan=2002-2015 |
| Campbell Collaboration  The Campbell Library | 1 | gambling or gamble* |
|  | 2 | GamAnon or "Gam Anon" or "Gam-Anon" |
| WorldCat | 1 | “Gamblers Anonymous” |
|  | 2 | GamAnon or "Gam Anon" or "Gam-Anon" = in keyword or title 2002-2015 |
