# Supplementary material for: Gamblers Anonymous as a Recovery Pathway: A Scoping Review
Source: J Gambl Stud. 2016 Apr 4;32(4):1261–78. doi: 10.1007/s10899-016-9596-8 (PMC5101261; doi:10.1007/s10899-016-9596-8)
Supplement: Supplementary file 2 — Supplementary material 2 (DOCX 29 kb) [file 10899_2016_9596_MOESM2_ESM.docx]

**Supplemental Table 2. Description of studies included (n =17)**^a^

| **Study ID** | **Associated studies** | **Country** | **Study design** | **Study aims** | **Number of participants** | **Age** | **Sex** | **MMAT Ratings** |
| --- | --- | --- | --- | --- | --- | --- | --- | --- |
| Linardatou 2014 |  | Greece | RCT | To examine the effectiveness of a stress management intervention as an adjunct treatment to GA, compared to GA alone on psychosocial functioning | 45 | Intervention: 43.2 [range 30-62]  GA: 42.0 [range 28-66] | Intervention: (21M:1F); GA (18M:2F) | *** |
| Desai 2012 |  | USA | RCT | To assess the effectiveness of bupropion and harm reduction, bupropion and GA, placebo and harm reduction, or placebo and GA on gambling frequency and intensity, treatment compliance, global functioning, disability, and treatment motivation | 36 | NR | NR | - |
| Grant 2009 |  |  | RCT | To compare the effectiveness of imaginal desensitization plus motivational interviewing (IDMI) to GA referral. | 68 | 48.7 (12.8) | 25M: 43F | ** |
| Petry 2006 | (Petry 2007; Ledgerwood 2007) | USA | RCT | To compare the effectiveness of a short-term, cognitive behavioural treatment to GA referral | 231 | GA referral: 44.4(11.7); GA + CB workbook: 44.3(9.4); GA +CBT: 45.8(11.6) | GA referral (30M:33F); GA + CB workbook (48M:36F); GA +CBT (49M:35F) | * |
| Petry 2003 |  | USA | cohort | To compare a sample of individuals entering professional treatment for pathological gambling who had experience with GA to those who had not previously attended GA | 342 | Non-GA attendees: 41.9(12.4); GA attendees: 44.4(10.7) | Non-GA attendees: 107M: 77F; GA attendees: 98M: 60F | **** |
| Oei 2008 |  | Australia | cross-sectional | To examine psychosocial factors that differentiated abstinent and relapsed individuals among GA members | 75 | 45(10) [range 21 - 64] | 55 M: 20 F | *** |
| Gomes 2008 |  | Canada | cross-sectional | To explore the change facilitating effects of certain factors (emotional support, instrumental support, emotional awareness, GA involvement, and depressed affect) in a sample of individuals being treated for problem gambling | 60 | 46.7 [range 18–70] | 33M: 27F | **** |
| Toneatto 2008 |  | Canada | cross-sectional | To report reliability and validity data for the Gamblers Anonymous Twenty Questions questionnaire in 3 independent samples of participants experiencing problems with gambling: two samples in treatment and one sample not receiving treatment | 456 | Treatment sample 1: 40.9 (14.7); Treatment sample 2:47.0 (14.2); Non-treatment sample: 43.4 (12.0) | Treatment sample 1: 39M: 22F Treatment sample 2: 73M: 26; Non-treatment sample: 130M: 166F | *** |
| De Castro 2005 |  | Brazil | cross-sectional | To report reliability and validity data for the Gambling Follow-Up Scale (GFS), a questionnaire assessing individuals recovering from gambling problems. A secondary goal included examining the associations between treatment type and the GFS | 47 | 44.7 | 28M: 19F | **** |
| Grant 2002 |  | USA | cross-sectional | To examine gender differences in participants with problem gambling issues in terms of demographic characteristics, gambling symptoms, and treatment history | 131 | Females: 48.4(9.6); Males: 46.6(12.9) | 53M: 78F | *** |
| Laracy 2012 |  | Canada | qualitative | To explore how individuals who play video lottery terminals (VLTs) perceive themselves and their gambling activity, the types of gambling-related stigma experienced by individuals who play video lottery terminals (VLTs), and management techniques used to cope with the label of “compulsive gambler” | 12 | 35-45 (N=7); 46-55 (N =3); 56-65 (N=1); 66 + (N = 1) | 7M:5F | **** |
| Ferentzy 2007 | (Ferentzy 2009; 2010a; 2010b) | Canada | qualitative | To explore common themes, as well as variations in recovery approaches among GA members | 39 | 56.5 [range 35-80] | 26M: 13F | **** |
| Straus 2006 |  | USA | qualitative | To explore the process and effects of direct member-to-member comments at GA meetings | 64 | NR | Survey sample: 37M:15F; Interview sample: 12M | ** |
| Ferentzy 2004 | (Ferentzy 2004; 2006a; 2006b) | Canada | qualitative | To provide an in-depth and empirically grounded account of GA's recovery culture | Observation of 42 GA and 29 NA meetings; 27 interviews (four NA members, 23 GA members) | Interview sample: [range 26 – 70] | Interview sample: GA: 15M: 8F; NA: 2M:2F | **** |
| Avery 2008 |  | USA | mixed methods | To identify recovery approaches and patterns specific to women | 136 | 18-24 (N = 3); 25-34 (N = 21); 35-44 (N = 39); 45-54 (N = 48); 55-64 (N = 16); 65+ (N = 5) | females only | * |
| Bulcke 2008 |  | USA | mixed methods | To explore the barriers to gambling treatment experienced by women | 90 | 47(13.9) [range 25 - 80] | females only | ** |
| Cooper 2003 |  | Canada | mixed-methods | To explore the use of computer-mediated communication as a recovery approach for individuals experiencing problems with gambling. The author also explored reasons individuals avoided self-help groups such as GA and/or specialist treatment services for gambling issues | 50 | 43.3 | 26M:24F | * |

^a^ Studies were scored as follows: – (0% of quality criteria met); * (25% of quality criteria met); ** (50% of quality criteria met); *** (75% of quality criteria met) or **** (100% of quality criteria met)
